# Construction Notes

**Published:** 1895-09-28

**Authors:** 


### CONSTRUCTION NOTES.

### Swansea Hospital.

It has been here deemed advisable to substitute copper pipes for defective lead ones now in use in the heating apparatus of the Talbot Ward and other wards. Mr. John Legge's tender of £105 for this purpose has been accepted.

### Glascow New Infectious Hospital.

The memorial stone of the new hospital for infectious diseases has just been laid at Glasgow. The site is on a breezy hill some 270 feet above the level of the sea, and is bounded on the two sides by the Forth and Clyde Canal. It occupies a space of 90 acres altogether. When completed, the entire hospital buildings are to be surrounded by a wall nine feet high, the main and only entrance being in Bilsland Drive—the single entrance being a special feature of the hospital to which much importance is attached as the simplest and most effective way of securing discipline. Inside the gateway there will be situated the houses for the detached officials, such as the house steward and the master of works. The carriage drive leads by the right direct to the administrative block, a building constructed of red sandstone, which will have architectural features of a sufficiently striking character to meet the requirements of a hospital intended to rank among the very first. Within this building is to be provided accommodation for the medical and nursing staff, including recreation-rooms and separate dormitories for 206 nurses. Behind this block the pavilions for the treatment of patients have been arranged. These consist of twelve large buildings, each to accommodate twenty patients, whilst each bed is allowed a minimum cubic measure of 2,000 feet. In addition there are four small pavilions intended for the accommodation of special cases of disease, and these will give accommodation for forty-eight cases. The lavatory and sanitary arrangements of all the pavilions are entirely detached from atmospheric connection with the wards, and generally the buildings have been constructed from plans prepared after utilising not only experience gained in the existing hospitals in Glascow, but in all the most recently constructed hospitals in Britain. In addition to the administrative and ward buildings there are to be large buildings erected for the dispensary, for the kitchen purposes, and for day workers. Washhouses, workshops, and stabling accommodation are also provided for, and in the centre of the buildings there will be a water tower. The estimated cost of the whole scheme is about £250,000. Dr. J. B. Russell, medical officer of the city, and Mr. A. B. McDonald, city engineer, are mainly responsible for the designing and internal planning of the hospital.

### Birmingham General Hospital.

Very satisfactory progress is being made with the new General Hospital, now in course of erection, with frontages in Steelhouse Lane, Loveday Street, Whittall Street, and Weaman Row. The four main portions of the new building, which will improve the neighbourhood in many ways, are now advanced to the height of the second storey, and with good fortune the contractors hope to have these pavilions roofed-in before the new year comes with inclement winter weather. The principal frontage of the building is in Steelhouse Lane, and the artistically-arranged terra-cotta facings already present a pleasant contrast to some of the dingy tenements around. Not much can be seen at present of the gardens, conservatory, and fountains which eventually will have the effect of converting an overcrowded district into what may be termed an open space in the centre of the city. From the work already performed a fair idea is to be had of what the general arrangement of the hospital will be, with its blocks of pavilions in the form of wings projecting at right angles from a corridor building of three storeys. At the front in Steelhouse Lane a handsome balcony or verandah is just commenced. This will overlook the spacious courtyard, with its fountain and gardens, and communicates by a French window with one of the largest of the wards. Near this entrance is the surgery. This room is arranged so as to communicate conveniently with the various wards, and as it is necessary to frequently remove a patient to a ward upon a higher storey a lift in the corridor outside the surgery is being erected. On the Loveday Street side more wards are being constructed, doctors' consulting-rooms, a room for the application of massage and electricity, operating and lecture theatres, and an ear and throat patients' ward, which will be fronted by another garden. At the back of the building are seen the outlines of retiring-rooms of the nursing staff and the servants' dining-rooms; and on the side bordered by Whittall Street will ultimately be the nurses' home, a building of three storeys, communicating with the general building by a covered way. This part of the building has at present not been commenced. On every floor of the hospital will be provided store-rooms. A thorough system of ventilation has been provided, and the method here in course of application is by Mr. William Key, of Glascow.

**New Infirmary for King's Norton.**

For some time past now the Guardians of King's Norton have been concerned about the accommodation at their disposal for the poor. After carefu l consideration, the Board decided that an infirmary is absolutely necessary. Plans for the erection of the same were accepted from Mr. Daniel Askell, of Birmingham. The building when completed will contain an administrative block in the centre, with medical officer's, matron's, and store-room, &c. The block is large enough to enable the officers to deal with an additional 250 patients, which, with the present provision, will give an accommodation for 500. On either side of the block will run corridors, which will be connected with the nurses' rooms, bath rooms, &c., with lavatories at the end, connected with short lobbies giving good ventilation. Emergency staircases will be provided in each ward, and hydrants will be placed in all wards in case of fire. The wards are so arranged that the air has free circulation, and they are so situated that they receive the full benefit of the sun, both from the east and from the west. In the rear of the administration block will be the cooking kitchens, nurses' and servants living rooms, culinary stores, wash-houses, &c., and will be sufficiently large in case of future extension of the infirmary. Special attention has been given to the sanitary arrangements, and the building is being constructed with a view to making it fireproof. The infirmary, although of a plain character, will have an imposing front, and the building, furnishing, &c., is estimated at £23,000.

